# Colonic metastasis of a huge soft uterine leiomyoma in a postmenopausal woman: a case report

**DOI:** 10.3389/fonc.2025.1660443

**Published:** 2025-10-28

**Authors:** JianXin Tang, RuiBo Xu, XiaoYu Xi, Ying Liu, ZengFang Hao, JieXian Du, XiaoLi Du

**Affiliations:** ^1^ Department of Gynecology, The Second Hospital of Hebei Medical University, Shijiazhuang, Hebei, China; ^2^ Department of Gynecology, Handan First Hospital, Handan, Hebei, China; ^3^ Department of Gynecology, Peking University People’s Hospital, Beijing, China; ^4^ Department of Pathology, The Second Hospital of Hebei Medical University, Shijiazhuang, Hebei, China; ^5^ Department of Gynecology, Shijiazhuang Hospital of traditional Chinese Medicine, Shijiazhuang, Hebei, China

**Keywords:** a huge uterine fibroids, myoma fusion, sigmoid mesentery metastasis, degeneration of uterine fibroids, benign metastasizing leiomyoma

## Abstract

**Background:**

.Uterine leiomyomas are the most common benign tumors of the female reproductive system, arising from the overgrowth of smooth muscle tissue within the uterus. Typically, they present as solid, spherical masses that are firmer than the surrounding uterine muscle layer. Onset generally occurs between the ages of 30 and 50, with these tumors often located in the uterine body or cervix. Some women may experience gradual tumor enlargement, accompanied by symptoms such as vaginal bleeding, lower abdominal pain, frequent urination, and constipation. In such instances, surgical treatment should be considered the first choice.

**Case summary:**

In this case, A 59-year-old woman presented with a large, soft, and irregularly shaped uterine leiomyoma, formed by the fusion of hundreds of smaller fibroids, which had metastasized to the sigmoid colon.Immunohistochemical analysis further demonstrated that benign uterine leiomyomas may present with morphological features, consistency, and an invasive growth pattern akin to malignant tumors.

**Innovations:**

Uterine leiomyomas are benign tumors that rarely metastasize; however, case reports have documented instances of distant metastasis.

**Conclusion:**

The purpose of this study is to report a case of a uterine leiomyoma with intestinal metastasis, analyze its rare characteristics and invasive growth pattern, and enhance the understanding of this tumor.

## Case presentation

**Figure 1:**
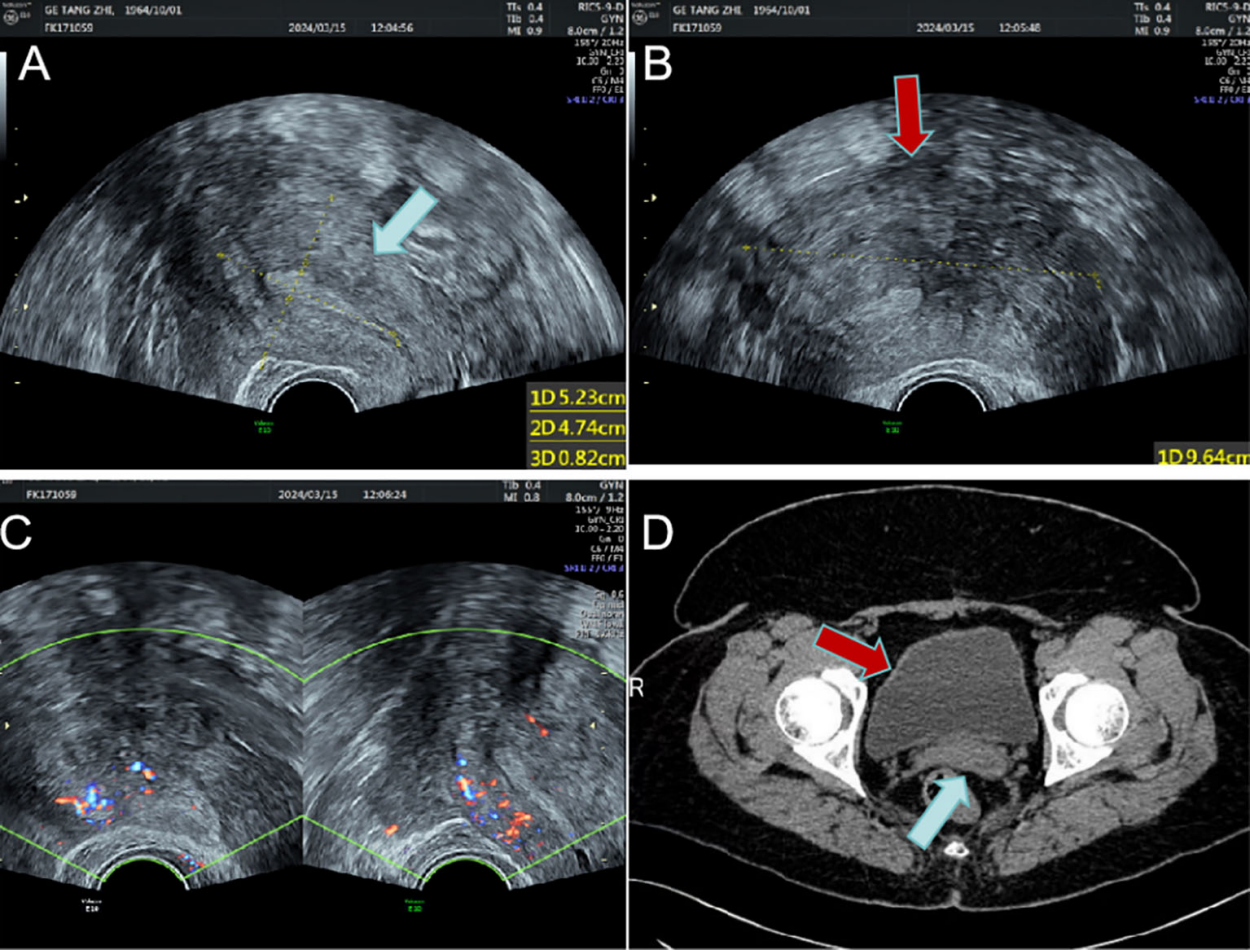
The uterus and the huge mass are shown on imaging. **(A)** Enlarged uterus on transvaginal sonography(blue arrow); **(B)** Large hypoechoic mass around the uterus(red arrow); **(C)** Blood flow signals around the uterus; **(D)** The uterus (blue arrow) and surrounding giant mass (red arrow) on enhanced CT.

**Figure 2:**
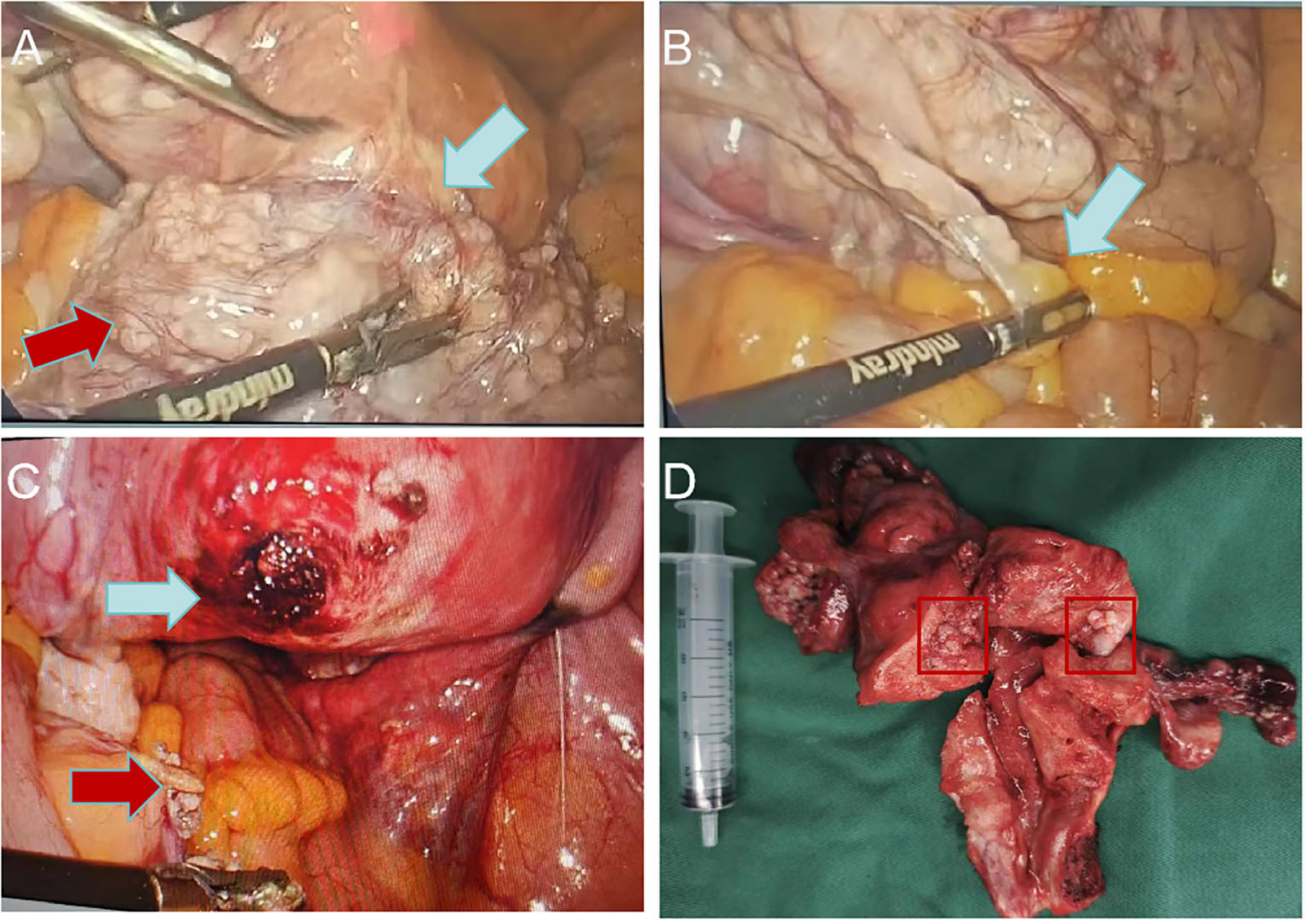
Laparoscopic uterine fibroids. **(A)** Uterine fibroids are large, soft, irregular in shape (red arrow) and originate from the posterior wall of the uterus(blue arrow); **(B)** Uterine fibroids invade the sigmoid mesentery (blue arrow); **(C)** Uterine fibroid pedicle (blue arrow) and sigmoid colon mesentery tumor after resection (red arrow); **(D)** Specimen of uterus.

A 59-year-old female was admitted to the hospital due to intermittent vaginal bleeding that had persisted for two years. The patient had been menopausal for two years and reported experiencing intermittent vaginal bleeding after menopause, occurring every 3 to 4 months. The bleeding was less voluminous than menstruation and lasted approximately three days each time. She also experienced lower back pain but did not report any lower abdominal pain or anal fullness. Physical examination revealed no abnormalities, and vaginal examination revealed an unobstructed vagina without mucosal congestion, a small amount of brown discharge, and no peculiar smell. The cervix was smooth, without bleeding, lifting pain, or rocking pain. The patient’s uterus and bilateral adnexa were not palpable due to the thick abdominal wall. Her medical history included diabetes and a tubal ligation performed 28 years ago, with no other significant abnormalities noted. The patient had a body mass index (BMI) of 35.2 kg/m². She had diet-controlled diabetes and denied taking any specific medications, especially estrogen-based agents. She also denied any history of smoking or alcohol abuse. The patient had a total of three pregnancies, which included one vaginal delivery and two miscarriages. Her family history was significant for digestive malignancies, with three immediate family members affected: two younger brothers diagnosed with gastric cancer and an older sister with pancreatic cancer. Liquid-based thin-layer cell testing revealed no intraepithelial or malignant lesions. Tumor four results showed AFP at 3.24 IU/ml, CEA at 1.84 ng/ml, CA-199 at 20.70 IU/ml, and CA125 levels at 46.70 IU/ml (normal range 0–35 IU/ml). Transvaginal ultrasound revealed a hypoechoic area around the uterus, and the tumor was poorly demarcated and the origin could not be determined (**Figures 1A–C**). Enhanced CT findings that the uterine myometrium showed uneven enhancement density with multiple small nodules, the largest about 1.8cm in diameter, and a surrounding area of low density measuring 18cm × 21cm with no enhancement (**Figure 1D**). The patient underwent laparoscopic exploration under general anesthesia. During the operation, the uterus was found to be enlarged as in the second month of pregnancy, irregular in shape, with a mass protruding from the posterior wall, about 18cm × 20cm × 20cm in size, which seemed to be composed of numerous tiny fibroids fused together and soft in texture. Between the tiny fibroids, they are connected by fibers (**Figure 2A**). Surprisingly, the tumor invaded the sigmoid mesentery (**Figures 2B, C**). Three fibroids were seen in the broad ligament of the left uterus, each about 4cm in diameter (**Figure 2D**). No abnormalities were found in the ovaries bilaterally. During the operation, the adhesions surrounding the pelvic mass were carefully separated, and the mass was removed from its pedicle. In consultation with the surgeon, the mass from the sigmoid colon was completely excised, and both specimens were sent for frozen section analysis. The analysis revealed the presence of a spindle cell tumor, suggestive of a uterine leiomyoma. After discussion with her family, a hysterectomy plus bilateral adnexectomy was finally performed, along with resection of the colonic tumor.

**Figure 3:**
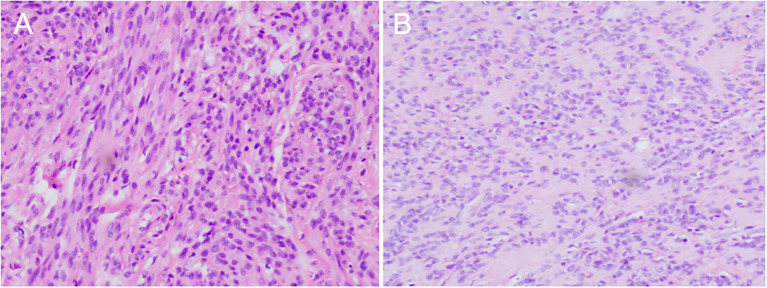
Hematoxylin eosin staining showed (magnifcation×200): **(A)** Large fibroids in the posterior wall of the uterus; **(B)** Metastatic lesions in the colon.

**Figure 4:**
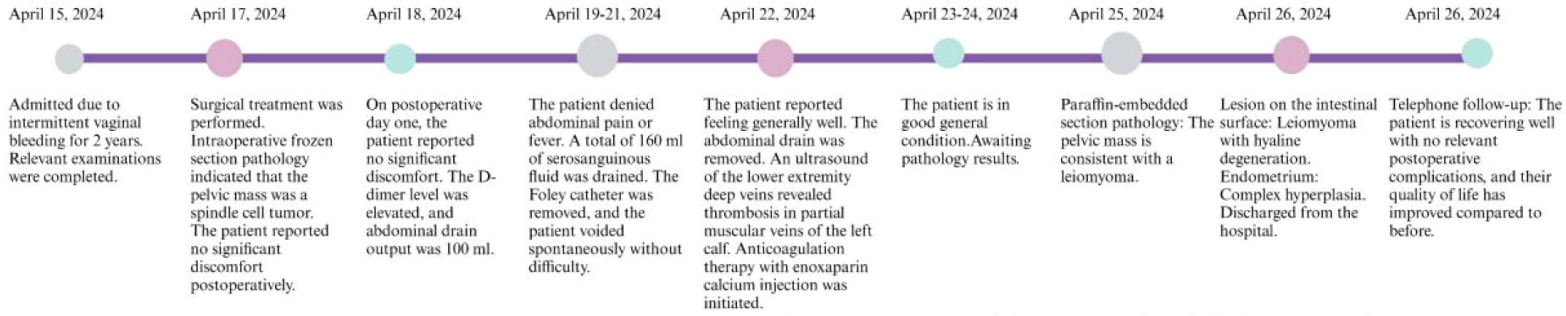
Clinical timeline of a rare leiomyoma presenting with intestinal metastasis.

**Figure 5:**
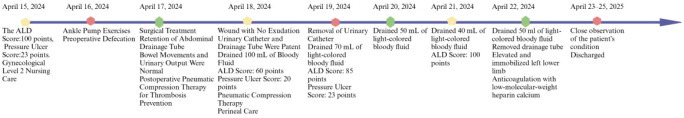
A timeline depicting the nursing events for a rare case of leiomyoma with intestinal metastasis.

Uterine and bilateral adnexal specimens: The uterus exhibited slight enlargement and irregular shape, with multiple small fibroids dispersed within the muscular walls. Additionally, three small fibroids were observed in the left lateral wall of the uterus, each appearing to be composed of numerous fused small fibroids. The endometrium was thick and smooth (**Figure 2D**). Combined with pathological morphology and immunophenotype, it is consistent with leiomyoma and immunohistochemistry analysis: calretinin (-), CD117 (-), CD34 (-), Desmin (+), DOG1 (-), Ki-67 (3%), SMA (+), SMMS-1 (+), α-inhibin (-) staining patterns (**Figure 3A**). Pathological examination of resected specimens from the uterus, bilateral adnexa, and intestinal surface revealed complex endometrial hyperplasia with eosinophilic metaplasia; endometrial polyps; glandular epithelial complex hyperplasia with eosinophilic metaplasia; myometrial multiple leiomyomas exhibiting edema, hyalinization, abundant cells in some areas along with occasional mitoses; chronic inflammation of the cervix; chronic inflammation of both fallopian tubes; inclusion cyst in the left ovary; while no abnormalities were found in the right ovary. The “colonic neoplasms” was leiomyoma with hyalinization. The results of immunohistochemistry showed that the endometrium: ER(+), HER2(1+), Ki-67(+5%), MLH1(+), MSH2(+), MSH6(+), P16(mottled +), P53 (no abnormal expression), PMS2(+), PR(+). Intermuscular leiomyoma: CD31 (vessel +)*2, CD34 (vessel +)*2, D2-40(-)*2, Desmin(+)*2, SMA(+)*2 (**Figure 3B**). Postoperatively, the patient experienced a successful recovery with stable vital signs, and no tumor recurrence was observed after surgery. Detailed information regarding the patient’s diagnosis, treatment, and nursing course is presented in **Figures 4** and **5**.

## Discussion

The origin of uterine leiomyoma can be attributed to the smooth muscle cells and fibroblasts within the uterus (1). These tumors often exhibit irregular shapes, firm textures, and are typically confined to the uterus without metastasizing to other organs or tissues (2, 3). Clinical manifestations commonly include abnormal uterine bleeding leading to anemia, pelvic pain, as well as compression symptoms such as frequent urination and constipation (4). Additionally, some patients may experience impaired fertility and pregnancy complications (5). The majority of uterine leiomyomas are hormone-dependent and typically exhibit growth cessation following menopause (6). However, in rare instances, they may persistently enlarge and metastasize distantly. In this case, we report an exceptional occurrence of a large soft uterine leiomyoma with sigmoidal metastasis in a postmenopausal woman. Notably, it is plausible that this variant of uterine fibroids shares a similar pathogenesis with benign metastasizing leiomyoma (BML).

The first reported case of benign leiomyoma with lung and mediastinal metastasis dates back to 1939 (7). To date, more than 150 cases of BML have been documented in the literature, yet the underlying mechanism remains elusive (8). Currently, no case of uterine leiomyoma metastasis to the sigmoid colon has been reported in the literature. It is postulated that this disease is influenced by sex hormones, which may induce specific alterations in genetics, transcriptome, and proteome profiles (9). These occurrences primarily involved women of reproductive age who had previously undergone myomectomy or hysterectomy without evidence of organ metastases at that time. Subsequently, metastases developed over time predominantly in the lungs and occasionally as pancreatic, mediastinal, or retroperitoneal masses (10–15) (**Table 1**).

**Table 1:** Cases of uterine leiomyoma with distant metastasis.

Case	Year of publication	Nationality	First author	Age(y ears)	Chief complaint	Uterine fibroids were single or multiple	The time of detection of uterine fibroid metastasis (preoperative, during the operation or after surgery)	Surgical methods	Site of metastasis	Clinical outcomes and follow-up
1	2020	USA	Luther B	46	dry cough	multiple	preoperative	Hysterectomy	Lungs, ovaries and abdominal wall	–
2	2021	Portugal	Diogo André	42	Hemoptysis	multiple	after surgery	Hysterectomy	Lungs and Ovaries	No recurrence
3	2020	Japan	Tsukasa Yoshida	53	Vaginal bleeding and cough	–	After surgery	Myomectomy	Lungs	No recurrence
4	2023	Japan	Naoki Minoda	59	Fever and headache	–	After surgery	Myomectomy	Pancreas	No recurrence
5	2019	China	Liqiang Huang	36	–	–	After surgery	Myomectomy	Lungs and greater omentum	No recurrence
6	2018	Vietnam	Nathaniel A. Parker	30	Flu-like symptoms and intermittent chest pain were noted	–	After surgery	Hysterectomy	Lungs	Persistence of the lesion
7	2018	Vietnam	Nathaniel A. Parker	33	Abdominal pain	single	After surgery	Hysterectomy and bilateral oophorectomy were performed	Lungs, heart and abdominal cavity	No recurrence

In contrast to other benign myometrial lesions (BML), it is noteworthy that the patient described in this medical record presented with uterine leiomyomas at two years postmenopausal, indicating a late age of onset. Furthermore, gynecological ultrasound and pelvic enhanced CT revealed a substantial hypoechoic lesion surrounding the uterus, which posed challenges in determining its nature. The morphological characteristics of this fibroid differed from those observed in other fibroids on imaging studies. Notably, the tumor exhibited diffuse growth patterns and lacked identifiable pedicle location, thereby impeding determination of its origin and nature. Consequently, laparoscopic exploration was performed following meticulous preoperative assessment. During the surgical procedure, it was observed that the tumor originated from the posterior uterine wall, exhibiting a soft and irregular texture with numerous smaller tumors conglomerated together. Moreover, these tumors were interconnected by fibrous tissues. Further exploration revealed that part of the tumor invaded the left mesentery of the sigmoid colon.

Because the tumor originates from the uterus, it exhibits a unique growth pattern and texture, necessitating differentiation from uterine sarcoma and advanced endometrial carcinoma. Uterine leiomyosarcoma and uterine leiomyoma share similar clinical features, as both arise from the myometrium and can present with symptoms such as abnormal uterine bleeding, pelvic pain, and the presence of a pelvic mass. However, a key distinction is that uterine leiomyosarcoma is invasive, has a very poor prognosis, and is prone to metastasizing to other sites. Some studies have indicated that rare histological and karyotypic variants of uterine fibroids may progress to uterine leiomyosarcoma (16). It is often challenging to distinguish uterine leiomyoma from uterine leiomyosarcoma through preoperative imaging examinations, and a definitive diagnosis is typically made post-surgery. Uterine sarcomas usually exhibit three histological features: multiple mitoses, severe cellular atypia, and coagulative tumor-cell necrosis, all of which were absent in this patient. Furthermore, it is essential to differentiate this patient from those with endometrial cancer, as advanced cases may penetrate the serosal layer and metastasize to other organs, often presenting with symptoms such as abnormal uterine bleeding (17). In most patients, this disease is suggested by preoperative gynecologic ultrasonography and MRI. Since the cancer originates from the endometrium, the lesion is typically concentrated within the uterine cavity. However, imaging of the patient reported in this case revealed that the tumor was primarily located outside the uterus, with no lesions observed in the endometrium. Surprisingly, we sent the tumor and the mass on the sigmoid colon for separate examinations, and the pathology results confirmed that the tumor originated from the uterus.

The patient is generally doing well following a total hysterectomy and is currently under regular follow-up with no evidence of recurrence. However, due to the specific nature of her tumor, we recommend that she undergo gynecological ultrasound examinations every six months, along with pulmonary CT, abdominal CT, and brain CT scans annually. During regular postoperative follow-up, examinations at 6 months—including ultrasonography and CT scans of the head, chest, and abdomen—demonstrated no evidence of metastasis. The patient reported a significantly improved quality of life attributable to the surgery, with no adverse or accidental events, and expressed high satisfaction with the treatment outcome. This approach aims to detect any signs of tumor metastasis to other organs as early as possible.

The possibility of a benign uterine leiomyoma should also be considered when tumors of uterine origin exhibit a similar shape, texture, and aggressive growth pattern. The necessity of intraoperative frozen section pathology lies in its utility to aid clinicians in determining the optimal scope of surgery. For postmenopausal patients, the preferred treatment modality is total hysterectomy plus bilateral adnexectomy. Should tumor metastasis to adjacent organs be identified, immediate excision by a multidisciplinary surgical team is imperative to inhibit further growth and safeguard organ functionality. Moreover, rigorous postoperative surveillance of these patients is pivotal. Pursuant to earlier BML studies, the incidental discovery of distant metastases in other organs post-surgery underscores the necessity for annual cranial, thoracic, and abdominal CT scans.

Due to limitations of the relevant instruments, we did not perform clonality testing, molecular analysis, or genetic comparison between the uterine and colonic lesions. Therefore, the findings may not fully prove that the sigmoid colon involvement represents genuine metastasis rather than direct extension or a parasitic leiomyoma. Further testing is required to confirm this pathological result.

## Conclusion

The discovery of uterine leiomyomas in postmenopausal women should be taken seriously. For this particular type of tumor, intraoperative frozen section pathology is essential, as it aids clinicians in making a preliminary assessment of the tumor’s nature and determining the appropriate scope of surgery. Although uterine leiomyomas are pathologically benign tumors, they can, in rare cases, exhibit malignant characteristics and invade surrounding organs. The preferred treatment is total hysterectomy, with the goal of removing the metastatic leiomyoma as completely as possible. Additionally, it is crucial for patients to be closely monitored after surgical treatment.

## Data Availability

The raw data supporting the conclusions of this article will be made available by the authors, without undue reservation.

## References

[1] Banerjee S Xu W Chowdhury I Driss A Ali M Yang Q . Human myometrial and uterine fibroid stem cell-derived organoids for intervening the pathophysiology of uterine fibroid. Reprod Sci. (2022) 29:2607–19. doi: 10.1007/s43032-022-00960-9, PMID: 35585291 PMC9444830

[2] Singh S Kumar P Kavita Rathore SS Singh Y Garg N . Contemporary approaches in the management of uterine leiomyomas. Eur J Obstetrics Gynecology Reprod Biol. (2023) 287:195–210. doi: 10.1016/j.ejogrb.2023.06.021, PMID: 37385088

[3] Harris HR Petrick JL Rosenberg L . The epidemiology of uterine fibroids: Where do we go from here? Fertility Sterility. (2022) 117:841–2. doi: 10.1016/j.fertnstert.2022.01.037, PMID: 35277259

[4] Ahmad A Kumar M Bhoi NR Badruddeen Akhtar J Khan MI . Diagnosis and management of uterine fibroids: current trends and future strategies. J Basic Clin Physiol Pharmacol. (2023) 34:291–310. doi: 10.1515/jbcpp-2022-0219, PMID: 36989026

[5] Giuliani E As-Sanie S Marsh EE . Epidemiology and management of uterine fibroids. Int J Gynecology Obstetrics. (2020) 149:3–9. doi: 10.1002/ijgo.13102, PMID: 31960950

[6] Alset D Pokudina IO Butenko EV Shkurat TP . The effect of estrogen-related genetic variants on the development of uterine leiomyoma: meta-analysis. Reprod Sci. (2022) 29:1921–9. doi: 10.1007/s43032-022-00911-4, PMID: 35414045

[7] Steiner PE . Metastasizing fibroleiomyoma of the uterus: Report of A case and review of the literature. Am J Pathol. (1939) 15(1):89–110.7., PMID: 19970436 PMC1965022

[8] Awonuga AO Shavell VI Imudia AN Rotas M Diamond MP Puscheck EE . Pathogenesis of benign metastasizing leiomyoma A review. Obstet Gynecol Surv. (2010) 65(3):189–95. doi: 10.1097/OGX.0b013e3181d60f93, PMID: 20214834

[9] MaChado-Lopez A Simón C Mas A . Molecular and cellular insights into the development of uterine fibroids. Int J Mol Sci. (2021) 22(16):8483. doi: 10.3390/ijms22168483, PMID: 34445194 PMC8395213

[10] Minoda N Tada T Takatani M Nakamura S Wani Y . Pancreatic metastasis of leiomyoma found 27 years after uterine fibroid surgery. Clin J Gastroenterol. (2023) 16:931–6. doi: 10.1007/s12328-023-01842-6, PMID: 37632657

[11] Yoshida T Nagao T Ozawa R Hida K . Benign metastasising leiomyoma with endometrial carcinoma, with a differential diagnosis of metastatic lung cancer. BMJ Case Rep. (2021) 14(4):e240922. doi: 10.1136/bcr-2020-240922, PMID: 33846186 PMC8048007

[12] André D Gouveia F Luís H Caldeira M Perneta F Gonçalves MJ . Benign metastasizing leiomyoma – a case of benign metastasis. JRSM Open. (2021) 12(12):20542704211064482. doi: 10.1177/20542704211064482, PMID: 34925863 PMC8674719

[13] Huang L Shi G Wang Q Guo Y Cong M . Pulmonary and mediastinum metastasis of uterine leiomyoma. Medicine. (2019) 98(49):e18276. doi: 10.1097/MD.0000000000018276, PMID: 31804368 PMC6919442

[14] Parker NA Dakhil CSR Dakhil SR Lalich D . MS-4: metastasis of benign leiomyomas outside the uterus. Kans J Med. 11(2):1–11., PMID: 29796157 PMC5962322

[15] Adair LB . CT findings of pathology proven benign metastasizing leiomyoma. Radiol Case Rep. (2020) 15:2120–4. doi: 10.1016/j.radcr.2020.08.052, PMID: 32944110 PMC7481490

[16] Yang Q Ciebiera M Bariani MV Ali M Elkafas H Boyer TG . Comprehensive review of uterine fibroids: developmental origin, pathogenesis, and treatment. Endocrine Rev. (2022) 43:678–719. doi: 10.1210/endrev/bnab039, PMID: 34741454 PMC9277653

[17] Berek JS Matias-Guiu X Creutzberg C Fotopoulou C Gaffney D Kehoe S . FIGO staging of endometrial cancer: 2023. J Gynecologic Oncol. (2023) 34(5):e85. doi: 10.3802/jgo.2023.34.e85, PMID: 37593813 PMC10482588

